# Squamous Cell Carcinoma of Lung Atypically Involving Heart: A Case Report With Literature Review

**DOI:** 10.14740/cr424w

**Published:** 2015-10-25

**Authors:** Tony Ete, Pravin Jha, Bhupen Barman, Animesh Mishra, Manish Kapoor, Amit Malviya, Vandana Raphael, Neel Kanth Issar

**Affiliations:** a Department of Medicine, North Eastern Indira Gandhi Regional Institute of Health and Medical Sciences, Shillong, Meghalaya, India; b Department of Cardiology, North Eastern Indira Gandhi Regional Institute of Health and Medical Sciences, Shillong, Meghalaya, India; c Department of Pathology, North Eastern Indira Gandhi Regional Institute of Health and Medical Sciences, Shillong, Meghalaya, India

**Keywords:** Squamous cell carcinoma, Metastasis, Left ventricle, Endocardium

## Abstract

Cardiac metastasis usually appears in patients with disseminated tumor disease. Involvement of heart in malignancy is generally underestimated and found to be in up to 25% of post mortem patients who had died of cancer. Cardiac involvement in metastases is usually uncommon; however, it may present with tachycardia, arrhythmia, cardiomegaly, heart failure, dyspnoea, hypotension, and pulsus paradoxus. Right side of heart is commonly known to be involved and the order of frequency of malignancies to metastasize to layers of the heart is pericardium, myocardium and endocardium.

## Introduction

Involvement of heart in neoplasm may be primary or secondary. Most common part of heart that is involved in metastases is pericardium. Involvement of endocardium is very rare. Cancers can metastasize to heart by different pathways including lymphatic, hematogenous, transvenous and direct route. Involvement of right side of heart in metastases is common. The most frequent neoplasms that metastasize to heart are melanoma, leukemia and lymphoma. Here we are reporting a case of squamous cell carcinoma of lung that metastasized to left side of heart involving the endocardium near the apical region.

## Case Report

**Figure 1:**
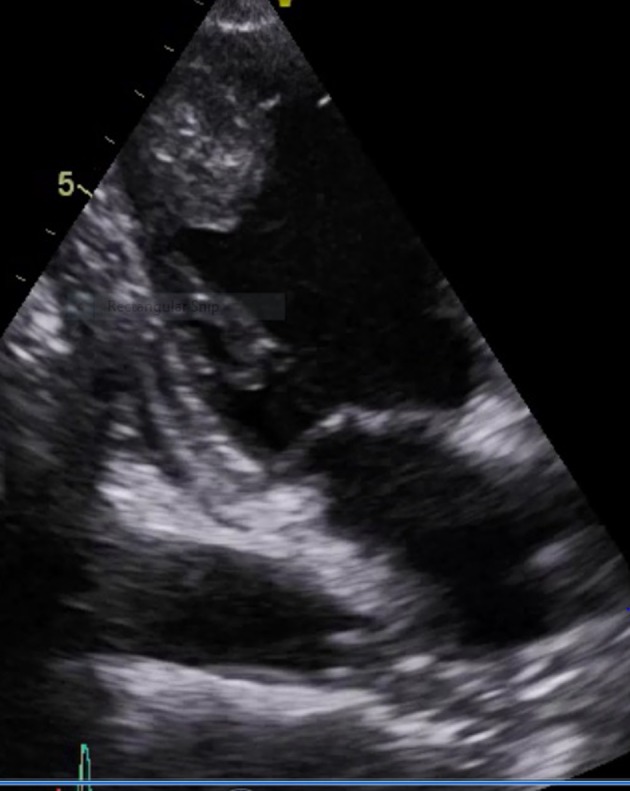
Apical four-chamber view showing heterogenous echogenic irregular vascular mass lesion in the endocardium with normal apical (left ventricular) mobility.

**Figure 2:**
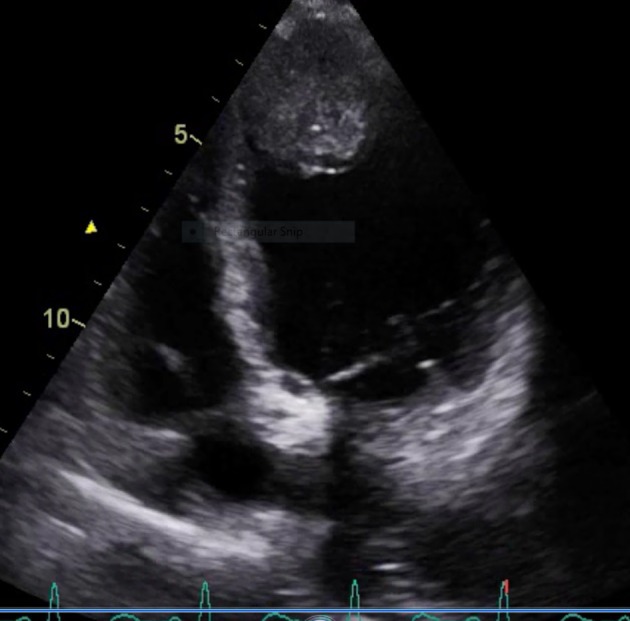
Apical two-chamber view showing heterogenous echogenic irregular vascular mass lesion in the endocardium with normal apical mobility.

**Figure 3:**
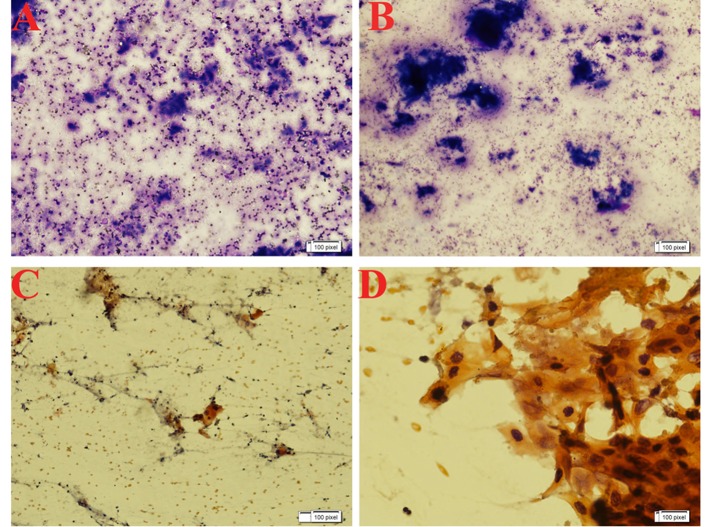
Maygrunwald Giemsa staining (A and B) and Pap staining (C and D) of the smears showing good cellularity consisting of malignant squamous cells arranged in tightly cohesive clusters and also dispersed singly. Occasional cells show individual cell keratinization. Background shows many degenerated cells necrosis and few cyst macrophages.

A 60-year-old male presented with cough for 6 months associated with decreased appetite and weight loss for the same duration. Cough was non-productive in nature with no history of any diurnal variation. There was no history of hemoptysis and any features suggestive of orthopnoea and PND. He also noticed sequential appearance of nodular swelling in left forearm, nasal tip and scalp. It was insidious in onset. Occasionally the patient had bleeding from nose. The patient had normal bladder and bowel habit. He was non-hypertensive, non-diabetic and had no history of contact with tuberculosis. On examination, higher mental function was within normal limit. Pallor and clubbing was present. Respiratory system revealed bronchial breath sound in right upper lobe. Cardiovascular system, gastrointestinal system and central nervous system examination revealed no abnormality. There was non-fluctuant, non-tender swelling in left forearm, scalp and nose. Laboratory investigations revealed Hb 10.2 gm%, total leukocyte count 11,000/mm^3^, platelets 1.8 lakhs/mm^3^, and ESR 70 mm/h. Renal function test and liver function test were within normal limit. Chest X-ray revealed a non-homogenous opacity in right upper lobe. Sputum for acid fast bacilli was negative on two occasions. Sonography of abdomen revealed no abnormality. Echocardiography showed an ejection fraction of 60%. A heterogenous echogenic irregular vascular mass lesion in the endocardium with normal apical mobility suggestive of secondaries in heart was seen (Fig. 1 and 2). Fine needle aspiration cytology from the left forearm mass and scalp swelling showed good cellularity consisting of malignant squamous cells in clusters, sheets and scattered singly in a background of giant cells, cellular debris, necrosis and blood elements suggestive of metastatic squamous cell carcinoma. Contrast-enhanced computerized tomography (CECT) of thorax was carried out along with CT-guided FNAC from the right upper lobe lung mass. Maygrunwald Giemsa staining (Fig. 3A, B) and Pap staining smear showed good cellularity consisting of malignant squamous cells arranged in tightly cohesive clusters and also dispersed singly. Occasional cells show individual cell keratinization. Background revealed many degenerated cells and few cyst macrophages suggestive of squamous cell carcinoma of lung (Fig. 3). The patient was diagnosed as a case of carcinoma of lung with secondaries to heart and multiple sites in skin.

## Discussion

Cardiac metastases usually occur in the sixth to seventh decade of life with no sex preference. Usually the diagnosis is made post-mortem. Most common malignancies that metastasize to heart are malignant melanoma, leukemia and lymphoma. However, the relative numbers are greater with breast and lung cancers [1]. Metastasis to heart is usually infrequent due to reasons like strong action of myocardium, rapid blood flow through the heart, metabolic peculiarities of striated muscle and lymphatic flow that is moving away from heart [2]. Usually it is the right side of the heart that is commonly involved during metastases than the left [3]. Morphologically, it is the pericardium that is most commonly involved in cancer compared to endocardium and myocardium. Neoplasms usually metastasize to heart by means of lymphatic, hematogenous route and direct extension, transvenous extension via the superior or inferior vena cava. Pericardial involvement usually involves lymphatic route and myocardial involvement involves hematogenous route. Endocardial involvement is rare. Metastasis to the heart is also seen quite often in adenocarcinoma of colon [4]. Carcinoma lung, which develops near heart, normally spreads to the heart via two mechanisms, either intracavitary diffusion via the pulmonary veins or direct extension preferentially involving the pericardium leading to pericardial effusion and other complications. Histopathologically, adenocarcinoma of lung spreading to lung has been documented [5]. Neoplasms that have metastasized to the heart are usually asymptomatic [6]. Retrospectively, only about one-tenth of patients who died of cancer and showed cardiac spread as identified at post mortem examination presented with symptoms or had findings indicative of cardiac involvement [7]. Clinical features are usually related to that of the disseminated organ involvement. Cardiovascular signs and symptoms include tachycardia, arrhythmia, cardiomegaly, heart failure, hypotension, dyspnoea, and peripheral cyanosis. The method of choice to detect cardiac metastases and their related complications is two-dimensional echocardiography [8]. Diagnosis of cardiac metastases can also be done through imaging techniques, i.e., CT or magnetic resonance imaging (MRI) [9]. Cardiac metastases are often incurable, so palliative therapy is offered. Systemic chemotherapy is usually the most beneficial. In our case, the primary tumor was squamous cell carcinoma of lung that metastasized to left side of the heart involving the endocardium in the apical region, clinically having tachycardia with no other cardiac manifestations, diagnosed echocardiographically which later was treated with palliative therapy.
